# The Glycoprotein/Cytokine Erythropoietin Promotes Rapid Alveolar Ridge Regeneration In Vivo by Promoting New Bone Extracellular Matrix Deposition in Conjunction with Coupled Angiogenesis/Osteogenesis

**DOI:** 10.3390/ijms22062788

**Published:** 2021-03-10

**Authors:** Mirali Pandya, Matthew Saxon, John Bozanich, Connie Tillberg, Xianghong Luan, Thomas G.H. Diekwisch

**Affiliations:** 1 Center for Craniofacial Research and Diagnosis, Texas A&M College of Dentistry, 3302 Gaston Avenue, Dallas, TX 75246, USA; pandya@tamu.edu (M.P.); ctillberg@tamu.edu (C.T.); xianghong.luan@tamu.edu (X.L.); 2 Department of Periodontics, Texas A&M College of Dentistry, 3302 Gaston Avenue, Dallas, TX 75246, USA; M.Saxon.dds@gmail.com (M.S.); appt1@santarosaperio.com (J.B.)

**Keywords:** alveolar ridge, erythropoietin, extracellular matrix, fibronectin collagen, bone regeneration, osteogenesis, angiogenesis

## Abstract

The loss of bone following tooth extraction poses a significant clinical problem for maxillofacial esthetics, function, and future implant placement. In the present study, the efficacy of an erythropoietin-impregnated collagen scaffold as an alveolar ridge augmentation material versus a conventional collagen scaffold and a BioOss inorganic bovine bone xenograft was examined. The collagen/Erythropoietin (EPO) scaffold exhibited significantly more rapid and complete osseous regeneration of the alveolar defect when compared to bone xenograft and the collagen membrane alone. The new EPO induced extracellular matrix was rich in Collagen I, Collagen III, Fibronectin (Fn) and E-cadherin, and featured significantly increased levels of the osteogenic transcription factors Runt-related transcription factor 2 (Runx2) and Osterix (Osx). Histomorphometric evaluation revealed a significant two-fold increase in the number of capillaries between the EPO and the BioOss group. Moreover, there was a highly significant 3.5-fold higher level of vascular endothelial growth factor (VEGF) in the collagen/EPO-treated group compared to controls. The significant effect of EPO on VEGF, FN, and RUNX2 upregulation was confirmed in vitro, and VEGF pathway analysis using VEGF inhibitors confirmed that EPO modulated extracellular matrix protein expression through VEGF even in the absence of blood vessels. Together, these data demonstrate the effectiveness of an EPO-impregnated collagen scaffold for bone regeneration as it induces rapid matrix production and osseoinduction adjacent to new capillaries via VEGF.

## 1. Introduction

The extracellular matrix (ECM) is an intercellular protein network that exerts profound control over cell function and behavior, directing cells to live or die, to proliferate, or to exit the cell cycle and differentiate [1]. During physiological organ function, the proteins and signals of the ECM are maintained in a tightly controlled state of homeostasis [2]. The ECM reveals its dynamic nature during development, tissue regeneration, or during pathological processes such as fibrosis, degeneration, or cancer, when cells deposit matrix or synthesize fibers to provide new environments for cells to grow. The ability of the ECM to form new matrix and tissues is frequently harnessed as a tool for tissue engineering, yielding remarkable similarities and parallels between ECM induced constructive remodeling and normal tissue morphogenesis [3]. ECM-based scaffolds are used for many tissue-engineering applications, including the repair and functional reconstruction of bone, skin, nerve, heart, lung, liver, kidney, small intestine, and other organs [4].

In bone tissue engineering, engineered scaffolds are designed to replace the natural bone matrix and also to provide templates for cell homing and mineralized tissue deposition. These engineered scaffolds mimic many of the properties of the natural bone matrix, such as cell proliferation and differentiation, cell adhesion and interactions, and the response to signaling stimuli. Engineered ECM-based scaffolds materials are frequently based on natural ECM components such as collagen, silk, chitosan or fibronectin, which are fabricated to display osteogenic, osteoconductive, or osteoinductive properties [5]. Collagen is the most abundant protein in the bone extracellular matrix and the most prominent extracellular matrix scaffold used for bone tissue engineering [6]. For tissue engineering purposes, collagen is frequently augmented with other factors to enhance its strength or augment its osteoinductive capacity [7].

Bone is a highly vascularized tissue [8]. During bone development, bone mineralization is tightly coupled with angiogenesis [9]. To form new bone, multipotent mesenchymal stem cells (MSCs) proliferate and differentiate into pre-osteoblasts, and then form pre-osteogenic condensations [10]. Prior to initial ossification, these condensations are invaded by small capillaries from surrounding tissues [11]. Afterwards, the earliest mineralized bone is associated with extensive internal and external vascularization [12]. The vasculature transports oxygen, nutrients, soluble factors and numerous cell types to the bone, facilitates osteogenesis, and maintains bone tissue integrity. For scaffold-based bone regeneration, blood vessels are also necessary to facilitate the inherent ability of stem cells to mobilize and to migrate to the scaffold surfaces where they will form new bone [13,14,15]. Successful bone regeneration after extraction of teeth provides opportunities for the rehabilitation of patients who have lost parts of their dentition.

Loss of alveolar bone following extraction of teeth is a common example of craniofacial bone loss with significant esthetic and functional consequences [16]. Extraction of teeth results in extensive remodeling of the vertical and horizontal dimensions of the adjacent alveolar ridge, largely due to reduction in strains and stresses exerted onto the mandibular bone [17]. The resulting loss of the jaw bone affects the long term stability of the jaw, the esthetic of the face, and a lack of suitability of the narrow bone ridge for subsequent implant placement [16,18].

As an alternative to freeze-dried bovine bone, allografts, alloplasts, or BMP-2 as alveolar ridge augmentation materials the kidney derived growth factor erythropoietin (EPO) was identified as a candidate molecule to enhance alveolar ridge augmentation in combination with a suitable scaffold. Currently, EPO overall safety in patients is established by current approval for Erythropoitin and biosimilars by the US Food and Drug Administration for the treatment of anemia caused by chronic kidney disease, chemotherapy, or use of zidovudine in patients with HIV infection. Even though there have been previous reports on the effect of EPO on bone healing [19], the mechanisms which are involved in the process of bone tissue restoration via erythropoietin are still poorly understood [20] and the applicability of EPO for scaffold-guided alveolar ridge augmentation is not known. The aim of the present study was to evaluate the efficacy of erythropoietin’s angiogenic and osteogenic potential compared to two popular ridge preservation techniques anorganic bovine bone mineral (Bio-Oss^®^) with non-cross linked collagen membrane (Bio-Gide^®^), and collagen membrane alone using a rat first molar extraction model [18], and to identify the extracellular matrix-related mechanisms by which EPO affects bone regeneration. To this end the efficacy of a combined collagen/erythropoietin scaffold to promote new formation of a highly vascularized novel bone matrix was determined.

## 2. Results

Application of EPO-soaked collagen scaffolds in rat molar extraction sockets resulted in rapid osseous regeneration after four weeks and continued mineralization in the 4–8 week interval.

**Figure 1:**
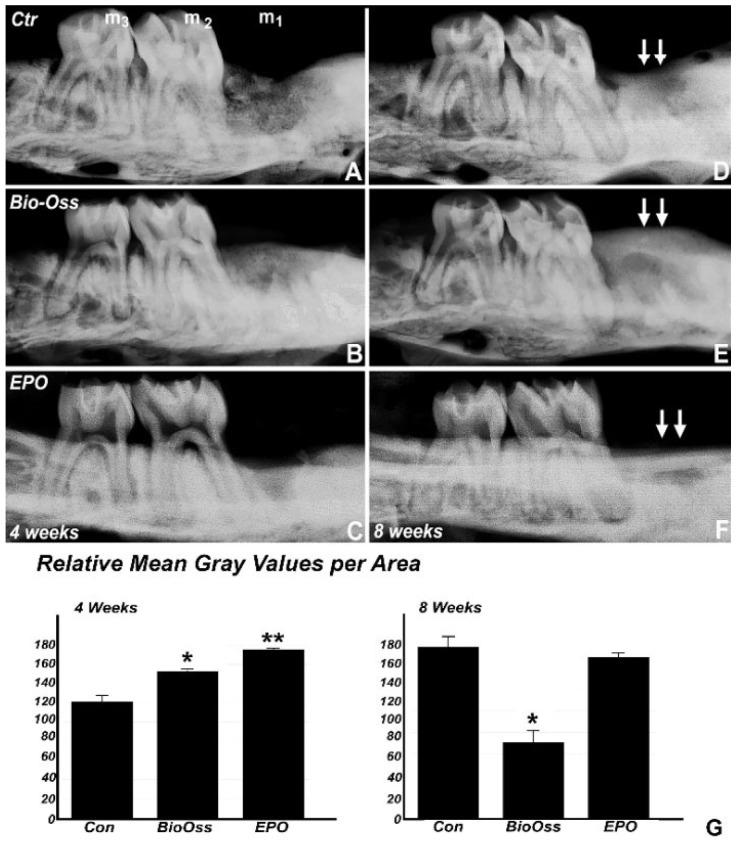
(**A**–**F**) X-Rays of 4 weeks (**A**–**C**) and 8 weeks (**D**–**F**) post-surgery rat maxillae following treatment with collagen membrane alone (**A**,**D**), BioOss (**B**,**E**) and EPO impregnated collagen sponge (**C**,**F**). Note the high radio-opacity in the EPO treatment groups 4 and 8 weeks after surgery (**C**,**F**) compared to the BioOss (**B**,**E**) and the collagen membrane control group (**A**,**D**). The graph in (**G**) revealed the significant difference in the mean grey value at 4 weeks indicated by * (*p* < 0.05) between the control and the Bio-Oss group and between the control and the EPO treated group (*p* < 0.01) indicated by **. At 8 weeks, the mean grey value was significantly lower in the Bio-Oss group compared to the control and EPO treated group (*p* < 0.05) indicated by *. The position of the rat maxillary molars is indicated as m_1_, m_2_, and m_3_, and the extraction site is demarked with a double arrow. The radio-opacity indicative of mineralization in the collagen/EPO treatment group was significantly higher than in the collagen membrane control group and in the Bio-Oss group 4 weeks after surgery.

Alveolar bone levels of rat maxillae four weeks and eight weeks post first maxillary molar extraction and after treatment with collagen membrane, Bio-Oss, and collagen/EPO were assessed by radiographs. Four weeks post extraction, the radiographs indicated that the alveolar socket in the collagen/EPO treatment group was almost completely filled with bone mineral and displayed a 48% higher level of mineralization than the control group, which still revealed detailed contours of the extraction socket. Mineralization levels in the Bio-Oss treatment group ranged between the collagen/EPO group and the collagen membrane control, with only a 27% increase compared to the control and outlines of the extraction socket still visible on the radiograph. The difference between the control and the Bio-Oss treatment group was significant, and the difference between the control and the collagen/EPO treatment group was highly significant after 4 weeks. After eight weeks, the control and the collagen/EPO treated groups displayed comparable levels of radiolucency, while the level of radio-opacity in the Bio-Oss group was significantly reduced. The radiograph once more demonstrated the most complete integration between the extraction site and the surrounding alveolar bone in the collagen/EPO treatment group suggestive of a highly successful osseous regeneration as seen in Figure 1.

The collagen/EPO scaffold promoted a significantly higher level of collagen I and III, fibronectin and E-cadherin extracellular matrix secretion when compared to the collagen membrane and the Bio-Oss group four weeks after implantation.

**Figure 2:**
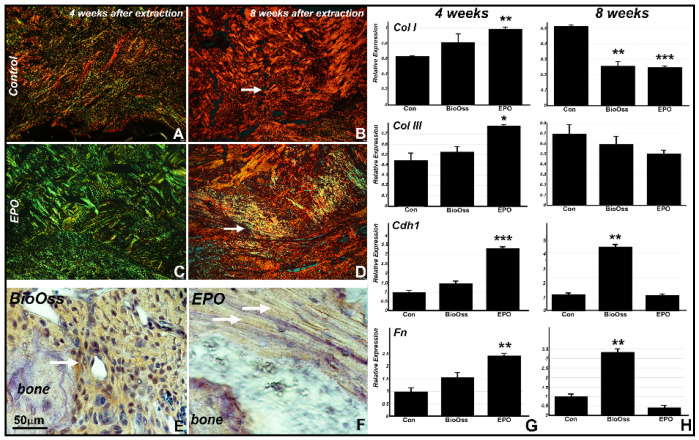
Picrosirius red staining (PSR) revealed well organized bundles of collagen fibers at the extraction socket site of the control and EPO treated groups of rats at 4 weeks (**A** and **C**) and at 8 weeks (**B** and **D**) post extraction. Immunohistochemical labeling demonstrating high levels of fibronectin protein expression in the newly formed extracellular matrix in the Bio-Oss group (**E**) and in sheath-like layers at the new bone formation sites in EPO treatment group (**F**). There was a significant increase in key extracellular matrix proteins collagen I, collagen III, E-cadherin 1, and fibronectin four weeks after surgery (**G**) and compared to BioOss and collagen membrane controls, while matrix protein production subsides eight weeks post-surgery (**H**). The significance value of *p* < 0.01 is indicated by ** and *p* < 0.001 is indicated by ***.

Picrosirius red staining was performed to study collagen fiber network organization in the four and eight weeks post extraction sites (Figure 2). Polarization microscopy suggested the presence of collagen I fibers 4 and 8 weeks post-surgery in the collagen membrane control group based on the consistently red birefringence in both groups, while there was a switch from green to red/yellow birefringence in the collagen/EPO treatment group suggestive of a change from collagen III to collagen I as the predominant matrix protein in the interval from 4 to 8 weeks post-surgery (Figure 2A–D). The significant effect of EPO on collagen III versus collagen I expression was confirmed in our in vitro studies (Figure 4C,D).

According to our real time RT-PCR analysis, all four matrix proteins investigated, collagens I (0.6 fold), III (0.25 fold), E-cadherin (2.2 fold), and Fibronectin (1.4 fold) were highly significantly upregulated at four weeks in the collagen/EPO scaffold group when compared to the collagen membrane control (Figure 2G). In contrast, the four matrix proteins studied here were either downregulated or unchanged in the collagen/EPO group eight weeks after surgery, while E-cadherin (3.5 fold) and Fibronectin (2.3 fold) were significantly upregulated in the Bio-Oss samples (Figure 2H). There was a peculiar difference in the organization of the newly formed fibronectin-rich matrix at the extraction socket site between the Bio-Oss and EPO groups as confirmed by immunohistochemistry: This new extracellular matrix in the collagen/EPO group was organized as a layer of extracellular matrix sheaths as compared to fibrous microstructure of the extracellular matrix surrounding the Bio-Oss scaffold (Figure 2E,F).

EPO caused Runx2/Osx mediated mineralized bone deposition coupled with a highly significant increase in VEGF expression and new capillary formation.

**Figure 3:**
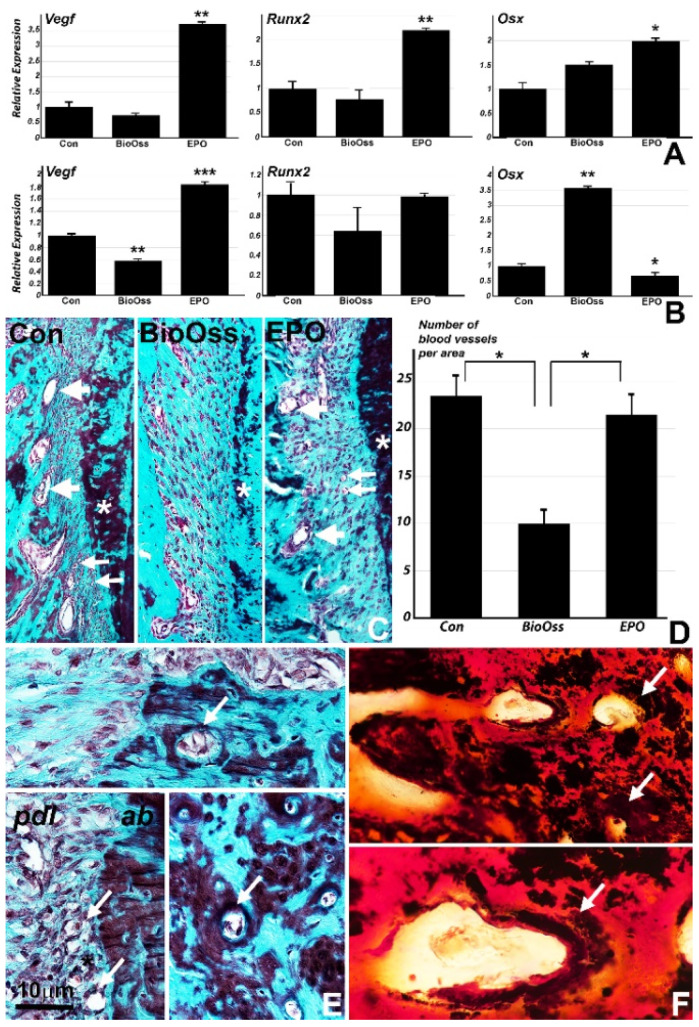
(**A**,**B**) Effect of collagen membrane, BioOss, and collagen/EPO scaffolds on the expression of key angiogenesis/osteogenesis genes four weeks after surgery (**A**) and 8 weeks after surgery (**B**). Note the significant effect of the collagen/EPO scaffold on Vegf and on the osteoblast transcription factors Osx and Runx2 (**A**). The significance value of *p* < 0.05 is indicated by *, *p* < 0.01 is indicated by ** and *p* < 0.001 is indicated by ***. There was a distinct presence of newly formed capillaries in the collagen/EPO treatment group when compared to the BioOss treatment group (**C**,**D**). E and F document a close proximity of the newly mineralized bone and blood vessels (small arrows) in the EPO treatment group as illustrated by trichrome stain (**E**) and von Kossa staining (**F**).

On a cellular level, osteoblastic lineage commitment toward mineralized bone formation is recognized by the key mineralization genes Runx2 and Osx [21,22]. In our alveolar bone defect model (Figure 3), gene expression for key mineralization genes Runx2 (1.3 fold) and Osx (1 fold) was significantly upregulated after 4 weeks in the collagen/EPO group when compared to the collagen membrane control and the Bio-Oss groups (Figure 3A). Eight weeks after surgery though, the Osx levels (2.5 fold) were significantly upregulated in the Bio-Oss group and significantly downregulated in the collagen/EPO group (0.4 fold) when compared to the control (Figure 3B). Vegf was significantly upregulated 4 weeks (2.2 fold) and 8 weeks (0.9 fold) in the EPO group while it was downregulated in the Bio-Oss group (0.4 fold) compared to the control (Figure 3A,B). The level of Vegf was 2.2 fold higher in the collagen/EPO group versus the control and the BioOss group after 4 weeks and remained twice as high 8 weeks after surgery, indicative of the significant effect of EPO on Vegf expression (Figure 3A,B). To determine whether there was a relationship between the high levels of Vegf and capillary formation or neovascularization, we determined the number of blood vessels per area using histomorphometry. Blood vessel counts were performed by comparing representative trichrome stained micrographs for all groups (Figure 3C,E) using ImageJ software. The number of blood vessels in the newly mineralized area were similar in the control and EPO group while they were significantly lower in the Bio-Oss group (Figure 3D). High magnification micrographs of eight weeks post-surgery alveolar regeneration sites demonstrated the close proximity between the blood vessels and the new ossification sites as revealed by von Kossa staining (Figure 3E,F, arrows).

EPO rescued the inhibitory effect of the VEGF inhibitor on matrix protein expression, demonstrating that VEGF is a major effector of EPO in terms of matrix secretion and bone formation.

**Figure 4:**
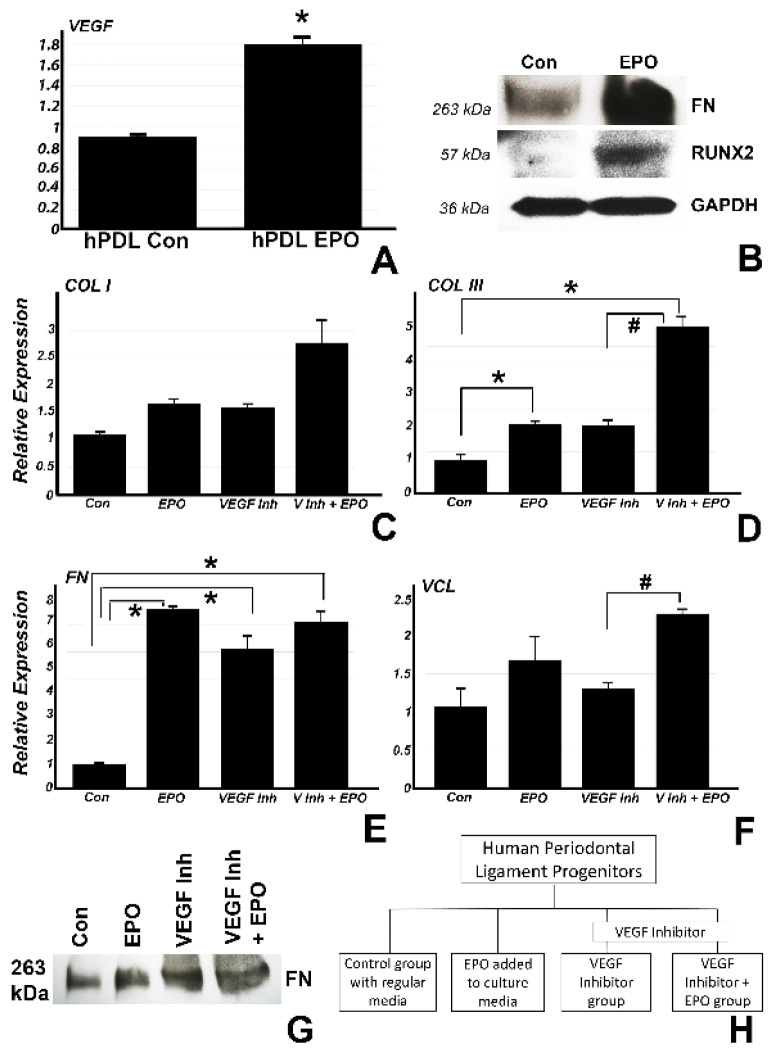
Relative Vegf gene expression in cultured hPDL cells was significantly upregulated following treatment with 0.5 µg/mL EPO for 1 week compared to the control group (**A**). Western blot analysis confirmed an increase in Fibronectin (FN) matrix protein and Runx2 osteoblast differentiation factor expression in the EPO group compared to the control (**B**). For pathway analysis studies, cultured hPDL cells were treated with control media, 0.5 µg/mL EPO, 10 µm VEGF inhibitor and VEGF inhibitor with EPO addition for 24 h (**C**–**H**) and the effect of treatment conditions on collagen I, III, fibronectin, and vinculin was assessed. In these studies, EPO treatment resulted in a significant upregulation of Collagen III (**D**) and FN (**E**) compared to the control confirming the capability of EPO to promote new mineralization and matrix formation. Inhibiting VEGF while adding EPO further upregulated the expression levels of collagen III (**D**), fibronectin (**E**) and for vinculin (**F**) in a significant fashion. The effect of EPO, VEGF inhibitor treatment, and EPO rescue on fibronectin protein expression was confirmed by Western blot (**G**). The statistically significant difference of *p* < 0.05 between an experimental group versus the control is indicated by an * and a statistically significant difference of *p* < 0.05 between two experimental groups is indicated by a #.

The effects of VEGF on neovascularization are well established [23], providing a plausible concept for the increase in capillary formation in collagen/EPO treated extraction sockets. In the present study, we asked the question whether VEGF may have other functions related to extracellular matrix synthesis based on the spike in new matrix proteins detected as a result of the collagen/EPO scaffold implantation (Figure 4). In a first set of studies, we demonstrated that vegf was significantly upregulated (0.8 fold) in vitro after addition of EPO to the culture media (Figure 4A), suggestive of an effect of EPO on VEGF even in the absence of blood vessels. In a similar experiment, EPO addition to periodontal progenitors (Figure 4B) demonstrated higher protein expression for FN and RUNX2 as compared to the control by Western blot, indicating the EPO directly affects matrix protein and mineralization gene expression without the presence of surrounding tissues or scaffolds. In a second set of in vitro studies. we determined the involvement of the VEGF pathway in EPO-mediated upregulation of matrix protein expression by employing a VEGF inhibitor. In this study, human periodontal ligament progenitors were cultured in vitro for 24 h with control media (group 1), 0.5 µg/mL EPO (group 2), 10 µM VEGF inhibitor (group 3) and 10 µM VEGF inhibitor with addition of EPO (group 4). Real time RT-PCR revealed a significant upregulation of COL III in groups 2 (1.2 fold) and 4 (4 fold) as compared to group 1 and an upregulation in group 4 (2.9 fold) as compared to group 3. FN was significantly upregulated in groups 2 (6.5 fold), 3 (5.1 fold) and 4 (6 fold) compared to group 1. There was also a significant upregulation for VCL in group 4 (1.25 fold) as compared to group 3. The effect on fibronectin protein expression was confirmed by Western blot analysis (Figure 4G).

## 3. Discussion

**Figure 5:**
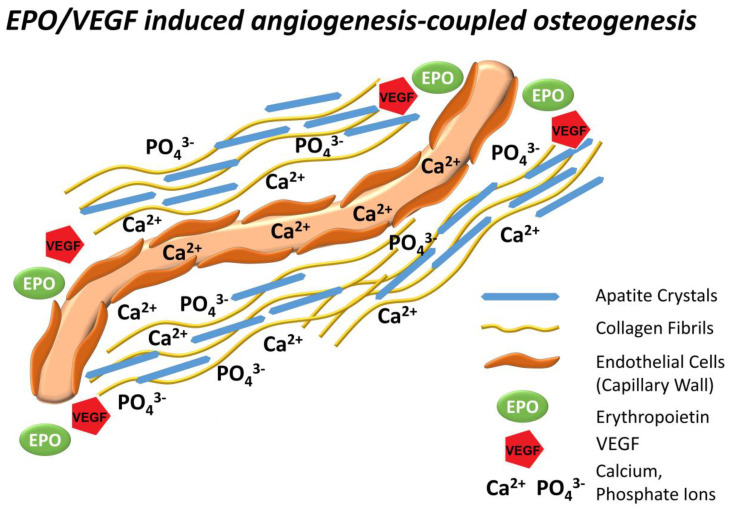
We hypothesize that EPO promotes new bone formation in an alveolar defect model by depositing a strong collagen I/III and fibronectin-rich extracellular matrix which upon VEGF induced new blood vessel invasion facilitates new calcium phosphate precipitation and apatite crystal growth.

The present study was based on a modified rat first molar extraction model [18] to demonstrate the efficacy of a erythropoietin-impregnated collagen sponge scaffold as an alveolar ridge augmentation material in comparison to inorganic bovine bone mineral (Bio-Oss^®^) with non-cross linked collagen membrane (Bio-Gide^®^), and collagen membrane alone. This study demonstrated rapid and complete osseous regeneration of the alveolar defect within four weeks after surgery and continuously increased levels of mineralization eight weeks after surgery using our EPO-impregnated collagen scaffold, significantly exceeding the outcomes of our collagen membrane control as well as the Bio-Oss xenograft conventionally used for alveolar ridge augmentation. It was anticipated that the alveolar defect in the EPO treated group was occupied by newly formed extracellular matrix, and our data demonstrated a significant increase in collagens, E-cadherin, and fibronectin. Corresponding to the increased levels of mineralization in the alveolar defect following EPO treatment, osteoblast transcription factors Runx2 and Osx were significantly elevated four weeks after surgery. In a quest to explain the rapid new bone formation and mineralization following collagen/EPO scaffold implantation, changes in Vegf expression and new capillary formation were examined, and both were significantly enhanced in the EPO treatment group versus the BioOss and the collagen control group. To ask whether EPO affects extracellular matrix secretion in a VEGF dependent fashion, it was verified whether EPO affects VEGF, Fibronectin, and Runx2 in vitro. Further, administration of VEGF inhibitors confirmed that EPO modulates the expression of collagen I, III, fibronectin, and vinculin matrix protein expression through VEGF even in the absence of blood vessels. Together, these data established that an EPO impregnated collagen scaffold served as an effective means for alveolar ridge augmentation, surpassing the regenerative potential of conventionally used bovine bone or collagen scaffolds. This study demonstrates that EPO functions by promoting new extracellular matrix secretion and increased biomineralization in conjunction with new capillary formation through VEGF (Figure 5).

Data presented in this study demonstrated that EPO promoted new extracellular matrix formation as evidenced by a significant increase in the extracellular matrix proteins collagen I and III, fibronectin, and E-cadherin. This new extracellular matrix was organized into sheath-like arrangements rather than in a physiological fashion of parallel organized and intercrossing collagen fibers. The effect of an EPO-impregnated collagen scaffold to form and organize a new extracellular matrix consisting of collagens, fibronectin, and other matrix proteins has not been previously reported. However, a previous study has reported that EPO promotes the assembly of a provisional extracellular matrix by recruiting β-integrin to the cell surface [24]. The deposition of a fibronectin-rich extracellular matrix as described here is destined to facilitate the binding of matrix components to α5β1 integrin to form fibrillar networks that will provide the structural and molecular foundation for new tissue organization [25]. In the present study, the elevation in matrix proteins was limited to the first four weeks after surgery and did not continue into the second four-week interval investigated here, suggesting that EPO results in a one-time burst of matrix protein secretion that does not continue over time. Such a one-time spike in secretion is desirable for any engineering factor to ensure that oncogenic side effects due to long term exposure to potential mutagens are kept to a minimum.

This study provided evidence that EPO caused Runx2/Osx mediated mineralized bone deposition coupled with a highly significant increase in VEGF expression and new capillary formation. While earlier reports have proposed that EPO induces osteogenesis by coupling hematopoiesis with bone induction signals [26] and that EPO promotes calvarial bone regeneration [19], the present paper demonstrates successful alveolar ridge engineering with the aid of a collagen/EPO scaffold. This study for the first time established a mechanism by which EPO promotes new bone formation as a combined effect of extracellular matrix deposition, mineralization gene upregulation, and VEGF pathway modulation. In terms of the effect of EPO on mineralization genes, our study demonstrates that both key osteoblast differentiation factors, Osx and Runx2, were approximately two-fold upregulated following EPO/collagen scaffold treatment, offering an explanation for the positive effect of EPO on bone matrix mineralization. In support of these findings, EPO administration alone upregulated both fibronectin and Runx2 in vitro, illustrating that the effect of EPO on matrix synthesis and mineralization was independent of the collagen scaffold and the surrounding environment.

These data demonstrate that EPO promotes new bone formation in an alveolar defect through several mechanisms related to EPO’s effect on VEGF upregulation. Data presented here have established that EPO triggered a highly significant five-fold upregulation of VEGF in an alveolar ridge regeneration model as well as a two-fold significant upregulation in vitro, confirming earlier studies about the effect of EPO on VEGF [27,28]. However, these studies have also demonstrated that the effect of EPO on new matrix protein expression is modulated through VEGF, as EPO rescued the effect of the VEGF inhibitor on collagen I, III, fibronectin, and vinculin expression. This finding suggests that EPO directly promoted new extracellular matrix synthesis through VEGF. Studies presented here also reveal a significant increase in new capillary formation and adjacent bone mineralization in the EPO treated group, suggesting that EPO promotes new bone formation through coupled angiogenesis/osteogenesis.

## 4. Materials and Methods

### 4.1. Animal Studies and Procedures

All animal procedures were approved by and were in compliance with the guidelines provided by the Texas A&M College of Dentistry IACUC committee (IACUC #2020-0074) and ARRIVE [29]. Study Design: Thirty ten weeks old male Sprague Dawley rats were used in the study. Rats were acclimated for one week in TAMU animal care facilities prior to the beginning of the experiments. First maxillary molars were extracted bilaterally and the rats were randomly assigned into three different groups (control, Bio-Oss, and collagen/EPO). Following tooth extraction, rats were fed dietary gel for three days and thereafter returned on a regular diet.

The rats weighed between 350–450 g and underwent general anesthesia via a mixture of ketamine (100 mg/kg)/xylazine (5 mg/kg). The right and left maxillary molars were extracted after careful elevation with a 7/8 Younger-Good Currette (Hu-Friedy^®^, Chicago, IL, USA). Next, a #703 fissure bur (Brasseler USA^®^, Savannah, GA, USA) was used in the extraction socket to create 3 mm deep, uniform defect. For the Bio-Oss group, the extraction sockets were preserved with inorganic bovine bone mineral (Bio-Oss^®^, Geistlich, Princeton, NJ, USA)) and for the EPO treated group, the extraction sockets received eukaryotic erythropoietin (EPO), 100 µg/mL (Biomatik^®^ RPU54825, ON, Canada) via a saturated Salvin^®^ OraPlug absorbable collagen sponge (Salvin^®^ Dental Specialties, Charlotte, NC, USA) (10 µg/mL final concentration). All sites were covered with a 1 × 1 mm resorbable collagen membrane (Bio-Gide, Geistlich Biomaterials), which was secured in place with periacryl purified cyanoacrylate dental adhesive (Salvin^®^ Dental Specialties). The rats were sacrificed after 4 weeks or 8 weeks of surgery.

### 4.2. Radiographs

Hemimaxillae from treated and control rats were fixed in 10% formalin for 5 days and analyzed with a Faxitron MX-20 specimen radiography system (Faxitron X-ray Corp., Aurora, OH, USA) at 20 kV for 20 s.

### 4.3. Paraffin Embedded Samples and Immunohistochemistry

All samples harvested from rats were fixed in 10% formalin for 5 days followed by decalcification in 10% EDTA for another 5 days in a precision pulsed microwave oven (Electron Microscopy Sciences, Hatfield, PA, USA). After decalicification, the samples were processed for regular paraffin sectioning after dehydrating through graded series of ethanol and xylene and cut into 5 µm thick sections. Immunohistochemical staining was performed based on the streptavidin biotin-peroxidase complex technique following the manufacturer’s instructions (Invitrogen, Carlsbad, CA, USA). Paraffin sections were deparaffinized, rehydrated and incubated in 10 µm sodium citrate buffer at 60 °C for 30 min for antigen retrieval. Briefly, after blocking endogenous peroxidase and non-specific antigens, the sections were incubated in anti-fibronectin primary antibody concentration 1:1000 (Ab2413; Abcam, Cambridge, UK) for 2 h at room temperature to detect the fibronectin protein localization and expression in rat tissues for the Bio-OSS and the collagen/EPO group. Isotype matched antibodies were used as negative controls. After washing in PBST, the sections were incubated with broad spectrum HRP conjugated broad spectrum secondary antibody supplied with the kit for 40 min at RT, followed by incubation with peroxidase conjugated streptavidin for 20 min at room temperature and washing with PBST. An AEC substrate kit (Invitrogen) was used to detect the immunoreaction, and the sections were counterstained with hematoxylin (Vector labs, Burlingame, CA, USA).

### 4.4. Ultrathin Ground Sections

Three hemi-maxillae from each group were fixed in 10% formalin and processed for ground section embedding as per the manufacturer’s protocol (EXAKT, Oklahoma city, OK, USA). After the samples were in 100% light cure technovit (Technovit 7200, EXAKT), the next step was to polymerize and embed the samples. Next, the samples were sectioned using a diamond bandsaw (EXAKT 300 CP), ground and polished to achieve 50–70 µm thin sections as described in our previous study [30].

### 4.5. Von Kossa Staining

Ultrathin ground sections were stained after undergoing rehydration, followed by incubation in 1% silver nitrate solution in clear glass under direct sunlight for 20 min. The ground section slides were rinsed in distilled water, and the excess silver ions were removed with incubation in 5% sodium thiosulfate for 5 min. The slides were then counterstained with nuclear fast red for 5 min. After rinsing with water again, the slides were dehydrated and mounted with a xylene based mounting medium.

### 4.6. Masson’s Trichrome Staining

Paraffin sample slides were rehydrated and stained with Weigert’s iron hematoxylin solution for 10 min, rinsed in warm tap water, washed in distilled water, stain with fuchsine solution and incubated with phosphomolybdic-phosphotungstic acid solution. Samples were then stained with aniline blue solution, rinsed with water and immersed in acetic acid solution. All procedures were carried out using a trichrome stain kit (Sigma-Aldrich, St. Louis, MO, USA). Sections were then quickly dehydrated and mounted using a xylene based mounting medium.

### 4.7. Picro-Sirius Red Staining

Paraffin sections were deparaffinized, rehydrated, and stained with Weigert’s hematoxylin for 8–10 min, followed by a wash and incubation in 0.1% Picrosirius red solution for 1 h. The slides were then washed in acidified water (0.5% hydrogen chloride) for 5 min, quickly dehydrated and mounted with a xylene based mounting medium.

### 4.8. In Vitro Culture of Human Periodontal Ligament Progenitors

For the first set of experiments, human periodontal ligament progenitors were cultured in vitro for one week. The cells were divided into two groups where one group was just supplied with regular DMEM medium while the second group had an addition of 0.5 µg/mL concentration of EPO along with the media. Fresh media was added every other day for both groups of cells. For the second in vitro experiment, the cells were cultured with regular media, 0.5 µg/mL concentration of EPO, VEGF inhibitor (EMD Millipore, Burlington, MA, USA; CBO-P11) and VEGF inhibitor with 0.5 µg/mL EPO for 24 h and harvested for RNA and protein extraction.

### 4.9. Western Blot

Whole cell lysates were used to extract proteins from cells using RIPA buffer with protease inhibitor (Roche, Basel, Switzerland) at 4 °C overnight. A Pierce BCA protein assay kit (ThermoScientific, Waltham, MA, USA) was used to measure the protein concentrations in each group. Proteins were separated via 10% SDS-PAGE gel and transferred to a polyvinylidene difluoride (PVDF) membrane using the semi-dry transfer system (BioRad, Hercules, CA, USA). The membrane was blocked with 5% non-fat dry milk powder for 1 h at RT and incubated with anti-GAPDH (GeneTex, Irvine, CA, USA; GTX100118) anti-Fibronectin (Abcam; ab2413), and anti-Runx2 (Abcam; ab23981) primary antibodies at 1:1000 overnight at 4 °C. After washing with TBST buffer, the membranes were incubated with anti-rabbit IgG HRP-linked (Cell signaling; 7074S) and anti-mouse IgG HRP-linked (Cell signaling, Danvers, MA, USA; 7076S) secondary antibodies accordingly at 1:2000 for 1 h at RT. After another wash with TBST buffer, the membranes were developed using a chemiluminiscent substrate (ThermoScientific).

### 4.10. RNA Extraction and Real Time PCR

RNA was extracted from rat maxillae frozen from 4 and 8 weeks groups and from the human periodontal ligament progenitors using RNeasy Plus kit (Qiagen, Hilden, Germany). For the RNA extraction from rat maxillae, the samples were first frozen using liquid nitrogen and immediately ground into powder form using a mortar pestle before proceeding to use the RNA extraction kit. One microgram of total extracted RNA was applied towards cDNA generation with RNA to cDNA ecodry premix (Takara, kusatsu, Japan). Real-time RT-PCR was performed using the SYBR green master mix, and sequence specific primers were designed for the study (Table 1) and CFX96 Real Time system (Bio-Rad). The reaction conditions were as follows: 2 min at 50 °C (1 cycle), 10 min at 95 °C (1 cycle), and 15 s at 95 °C, and 1 min at 60 °C (40 cycles). Samples were normalized against GAPDH. To quantify the relative differences in mRNA expression, the standardized comparative CT method (ΔΔCT) was used to determine relative quantity. All values were graphed as the relative mean expression level ± standard deviation. The gene expression for the in vivo rat samples was confirmed for Collagen I (Col I), Collagen III (Col III), E-cadherin (Cdh1), Fibronectin (Fn), Vegf, Runx2 and Osx for the rat samples. For the in vitro samples, gene expression was confirmed for COL I, COL III, FN, VINCULIN (VCL), and VEGF.

### 4.11. Statistical Analysis

The alveolar bone levels and mean grey values for the extraction socket site were compared across representative radiographs for each group using the ImageJ software following a previously published method [31]. The relative mean expression for the genes for the BioOss group and the collagen/EPO group were compared to the control group and for the in vitro relative gene expression data, each sample was compared across all groups. To compare the number of blood vessels across groups, representative micrographs at 10x magnification were used to count the blood vessels in the newly mineralized bone area using the ImageJ software. Statistical significance was assessed using ANOVA, and the significance level was set at *p* < 0.05.

## 5. Conclusions

Together, these studies have demonstrated unexpectedly efficient and rapid new bone formation following application of an EPO impregnated collagen scaffold. The effect of the EPO/collagen scaffold is explained as the result of a VEGF-mediated upregulation of extracellular matrix synthesis in combination with capillary ingrowth and coupled mineralization through increased expression of the osteoblast transcription factors Runx2 and Osx. Data presented here have demonstrated that the collagen/EPO scaffold introduced in this study results in the formation of an engineered bone extracellular matrix template that in conjunction with osteogenic factors and neovascularization harbors novel opportunities for the treatment of mid-size and large-size bone defects.

## Figures and Tables

**Table 1:** RT-PCR primer sequences.

**Rat RT PCR Primer Sequence**	**Forward**	**Reverse**
Col I a1	TCCCTATCCCTACCCTCAGCTTCTCT	AGTCTCTTGCTTCCTCCCAC
Col III a1	GTCCACAGCCTTCTACACCT	CGCCATTTCTCCCAGGAATG
Cdh1	ATCGCCTACACCATCCTCAG	GGGTAACTCTCTCGGTCCAG
Fn	CCGAATCACAGAT CGGTGAC	ATAGTCAATGCCGGGT TCCA
Vegf	CTCTCTCCCAGATCGG TGAC	CAAAGGAATGTGTGG TGGGG
Runx2	AGTTGGCTCTCATCCTTCCC	GCTGCTCCCTTCTGAACCTA
Osx	GAAGCGACCACTTGAGCAAA	ATTGGCTTCTTCTTCCCCGA
Gapdh	CAAGTTCAACGGCACAGTCA	CCCATTTGATGTTAGCGGG
**Human RT PCR Primer** **Sequence**	**Forward**	**Reverse**
COL I A1	CATCTCCCCTTCGTTTTTGA	CCAAATCCGATGTTTCTGCT
COL III A1	GATCAGGCCAGTGGAAATGT	GTGTGTTTCGCAACCATC
FN	TGGCACTGATGAAGAAC CCT	TGCCTCCACTATGACGTTGT
VEGF	CTCATCCTCTTCCTGCT CCC	CTCACACACACACAACCAGG
VCL	GTGCCTAGGCGCA TTTCA	GCTGCTCCCTTCTGAACCTA
GAPDH	ACAGTCAGCCGCATCTTCTT	ACGACCAAATCCGTTGACTC
